# Cigarette smoking and all-cause mortality in rural Chinese male adults: 15-year follow-up of the Anqing cohort study

**DOI:** 10.1186/s12889-021-10691-2

**Published:** 2021-04-09

**Authors:** Lijing Ye, Jie Yang, Jingyi Li, Nannan Cheng, Yue Zhang, Xiaofan Lu, Ziyi Zhou, Zhuo Wang, Lishun Liu, Xiao Huang, Yun Song, Shibo Xing, Dongqing Wang, Junnong Li, Binyan Wang, Genfu Tang, Xianhui Qin, Pierre Zalloua, Huisheng Zhang, Fangrong Yan, Xiping Xu

**Affiliations:** 1 State Key Laboratory of Natural Medicines, Research Center of Biostatistics and Computational Pharmacy, China Pharmaceutical University, Nanjing, 210009 China; 2 Beijing Advanced Innovation Center for Food Nutrition and Human Health, College of Food Science and Nutritional Engineering, China Agricultural University, Beijing, China; 3 Department of Cardiovascular Medicine, Second Affiliated Hospital of Nanchang University, Nanchang, China; 4 Institute of Biomedicine, Anhui Medical University, Hefei, China; 5 Health Center of Dongguo Center, Tengzhou, Shandong China; 6 Panjin People’s Hospital, Shenyang, Liaoning China; 7 Weinan Central Hospital, Weinan, Shanxi China; 8 Shenzhen Evergreen Medical Institute, Shenzhen, China; 9 School of Health Administration, Anhui Medical University, Hefei, China; 10 National Clinical Research Study Center for Kidney Disease; The State Key Laboratory for Organ Failure Research; Renal Division, Nanfang Hospital, Southern Medical University, Guangzhou, China; 11 School of Medicine, Lebanese American University, Byblos, Lebanon; 12 Guangdong Key Laboratory for Biomedical Measurements and Ultrasound Imaging, School of Biomedical Engineering, Health Science Center, Shenzhen University, Shenzhen, China

**Keywords:** Smoking status, Mortality, Chinese rural area

## Abstract

**Background:**

According to the Global Burden of Disease Study 2017, smoking is one of the leading four risk factors contributing to deaths in China. We aimed to evaluate the associations of smoking with all-cause mortality in a Chinese rural population.

**Methods:**

Male participants over age 45 (*n* = 5367) from a large familial aggregation study in rural China, were included in the current analyses. A total of 528 former smokers and 3849 current smokers accounted for 10 and 71.7% of the cohort, respectively. Generalized Estimating Equations were used to evaluate the association between baseline smoking status and mortality, adjusting for pertinent covariates.

**Results:**

There were 579 recorded deaths during the 15-year follow-up. Current smokers (odds ratio [OR],1.60; 95% CI,1.23–2.08) had higher all-cause mortality risks than nonsmokers. Relative to nonsmokers, current smokers of more than 40 pack-years ([OR],1.85; 95% CI,1.33–2.56) had a higher all-cause mortality risk. Compared to nonsmokers, current smokers who started smoking before age 20 ([OR],1.91; 95% CI,1.43–2.54) had a higher all-cause mortality risk, and former smokers in the lower pack-year group who quit after age 41 (median) ([OR],3.19; 95% CI,1.83–5.56) also had a higher risk of death after adjustment. Furthermore, former smokers who were also former drinkers had the highest significant risk of mortality than never smokers or drinkers. (*P* for interaction = 0.034).

**Conclusions:**

This study provides evidence that current smokers and former smokers have a higher mortality risk than nonsmokers and would benefit from cessation at a younger age.

**Supplementary Information:**

The online version contains supplementary material available at 10.1186/s12889-021-10691-2.

## Background

Smoking is a leading, but avoidable, cause of premature deaths and disability globally [1], contributing to an estimated 6 million deaths worldwide in 2010, including 1 million in China. The World Health Organization projects that by 2030 tobacco-attributable deaths will annually account for 3 million deaths in industrialized countries and 7 million in developing countries. China now consumes about 40% of the world’s total cigarettes, predominantly by men, with a large increase in consumption occurring in urban rather than rural areas over the past three decades [2–4]. During the past 20 years, a slight decrease in smoking prevalence was observed in the general Chinese population, however the rate of smoking cessation was only 20.1%, showing no significant change since 2010 [5]. An awareness of the harms of cigarette smoking and the diseases it can cause showed no obvious change from the cessation rates. Despite tobacco control, cigarette use remains the leading cause of premature mortality in China and globally. The 2017 Global Burden of Diseases, Injuries, and Risk Factors Study (GBD) estimated that high systolic blood pressure, smoking, and a diet high in sodium were the top three risk factors for number of deaths in 2017 in China. They were also the leading risk factors for disability-adjusted life-years (DALYs) in China overall and in 21 provinces, and were either second or third in all remaining provinces [6].

The hazards of smoking have been documented over the past 55 years, providing sufficient evidence of a causal relationship between smoking and many types of death [7]. A total of 160,113 participants of the NIH-AARP Diet and Health Study aged > 70 years showed that relative to never smokers, current smokers were more likely to die during follow-up (hazard ratio, 3.18; 95% CI,3.04–3.31) [8]. For participants who were 25 to 79 years of age in the U.S. National Health Interview Survey, the rate of death from any cause among current smokers was about three times that of those who had never smoked (hazard ratio for men, 2.8; 99% CI, 2.4–3.1) [9]. Early initiation of smoking is also related to increased mortality from all causes and disease-specific causes, such as vascular diseases (cardiovascular, coronary artery disease, cerebrovascular disease); respiratory diseases; and cancers [10]. In addition, the mortality risk associated with smoking cessation decreased compared with continuing smokers after 12 years of follow-up in the Nurses’ Health Study [7].

The China Health and Nutrition Survey (CHNS) study indicated that the current smoking rate in rural villages has remained relatively stable across the past two decades from 2000 (about 50–55%). The rate in urban neighborhoods decreased from 1991 to 2009, and then slightly increased to 25% in 2011 [11]. Residents living in socially and economically developed regions were less likely to smoke than those living in remote and underdeveloped regions. Those with lower levels of education and engaging in farm work were also more likely to smoke [12]*.* The urban population’s awareness of the above three diseases caused by smoking was higher than that of rural populations, with significant differences.

In the past two decades, the rate of cigarette smoking in rural areas has remained stable (nearly 50%), but it has fallen by approximately 1.1% annually in urban communities. After adjusting for individual and community characteristics, smoking prevalence shows few differences, statistically [11]. Trends in cigarette smoking are a signal of health inequalities. The common reasons for quitting include existing medical concerns, prevention of future health problems, family stress, and financial considerations [13]. Few people choose cessation under the advice of media advertisements or slogans. Among some mainstream media, over one- third of men reported not having paid attention to any health warnings about smoking hazards; these trends are higher in the elderly, in rural populations, and in those with lower education [4]. Traditionally, cigarette consumption for gifting and sharing during New Year’s Festival remains prevalent in rural neighborhoods. Among current and former smokers, sharing cigarettes is a major obstacle to quitting [13].

To date, most studies of cigarette smoking and mortality have focused on all-aged populations in the US, Australia and Korea [8, 14–16] with few studies examining the impact of tobacco use on disease and mortality risk among the middle-aged and elderly in China, especially in rural areas. To address this lack of evidence, we aimed to examine the association between smoking status with all-cause mortality among smokers and non-smokers using data from an osteoporosis cohort, which enrolled participants from Anqing, Anhui province, a rural area in Eastern China, in 2003.

## Methods

### Study design and participants

This study is part of a large community-based cohort initiated in 2003 among residents of Anhui Province, China [17, 18]. The major exclusion criteria included history of type 1 diabetes; renal failure; chronic infections such as tuberculosis or other infectious diseases; malignancies; rickets or other metabolic bone diseases; chronic glucocorticoid use; viral cirrhosis; and thyrotoxicosis. The questionnaire as the main tool to measure the smoke status in this community-based osteoporosis study has been previously published [17–19].

A total of 18,237 adults who participated in the baseline study were re-surveyed with a mean follow-up interval of 14.1 years. Eight thousand nine hundred ninety-five female participants were excluded from this analysis because the number of current and former smokers in the female population was too small to allow for regression analysis. After excluding participants with missing data on smoking status, pack-years of cigarette smoking, family numbers and anyone whose age was below 45 years, the final cohort consisted of 5367 male participants. All smokers were cigarette smokers (Supplemental Figure 1).

This study was approved by the Institutional Review Boards of Anhui Medical University. Written, approved, informed consent was obtained from each participant. The data supporting the findings of this study will be available from the corresponding author (Xiping Xu) on request.

### Death outcome collection

Follow-up visits with interviews and data collection were conducted in 2010, 2011, 2014, 2017, and 2018. Data on death was obtained by telephone or face-to-face interviews with participants or household members (if deceased).

### Smoking status, smoking intensity, age of smoking initiation and cessation age

Smoking status was self-reported by respondents and coded into three categories: current, former, and non-smokers. Current smokers reported smoking more than 10 packs in their lifetime and currently smoking every day or most days. Former smokers reported having smoked 10 packs or more cigarettes in their lifetime but currently having ceased. Non-smokers reported smoking fewer than 10 packs in their entire life. Current smokers were further disaggregated by pack-years [20] using the equation:

$$ \mathrm{Pack}-\mathrm{years}=\left(\mathrm{Cigarettes}\ \mathrm{per}\ \mathrm{day}/20\right)\ast \mathrm{years}\ \mathrm{smoked} $$

Pack-years was further divided into three categories (< 20, 20–40, ≥40 pack-years) [21], and as a binary variable where it was divided at the median for analysis. We disaggregated former smokers by years since age of smoking cessation (<55, and ≥ 55 years), and age of smoking initiation (for current smokers) was divided into two groups (<20, and ≥ 20 years).

### Statistical analysis

Means (SD) or medians (25th percentile-75th percentile) and proportions were calculated for population characteristics by smoking status. Differences between groups were achieved with analysis of variance. In the multivariate models, we adjusted for age, body mass index (BMI), systolic blood pressure (SBP), diastolic blood pressure (DBP), drinking status, fasting glucose (GLU), total cholesterol (TC), triglycerides (TG), high-density lipoprotein cholesterol (HDL-C) education level and occupation using Generalized Estimating Equations (GEE) to evaluate the association between baseline smoking status and mortality. Variables in the stratified analysis included BMI (Tertiles: < 20, 20–21.9, ≥20 kg/m^2^), age (: < 51.3, ≥51.3 years), SBP (< 130, 130–140, ≥140 mmHg or history of hypertension), DBP (< 80, 80–90, ≥90 mmHg or a history of hypertension), and drinking status (never, former, current).

A two-tailed *P* < 0.05 was considered statistically significant in all statistical analyses. EmpowerStats (http://www.empowerstats.com) and RStudio software (Version 1.2.5033, http://www.R-project.org/) were used for all statistical analyses.

## Results

### Study participants and baseline characteristics

Baseline characteristics of the participants according to smoking status (nonsmoker, former smoker and current smoker) are summarized for all males in Table 1. During the 15-year follow-up, the mean ages were 52.3(mean)4.8(SD), 52.5(mean)4.6(SD) and 52.0(mean)4.6(SD) for nonsmokers, former smokers, and current smokers, respectively. We found that current smokers tended to be younger and had lower blood pressure, body mass index, glucose, total cholesterol and triglycerides levels compared with nonsmokers (Table 1). The results were not similar in women due to the low number of current smokers. Of the 8995 women enrolled in our study, only 2.4% were current smokers, including 0.2% who stopped (Supplemental Table 2).

**Table 1:** Baseline characteristics of the study participants by smoking status in male

Variables	Smoking Status			***P*** value	***P*** value*
	Never (*n* = 990)	Former (*n* = 528)	Current (*n* = 3849)		
**Age, y**	52.3 (4.9)	52.5 (4.6)	52.0 (4.6)	0.023	0.154
**SBP, mmHg**	126.7 (19.8)	128.3 (21.2)	124.2 (19.6)	< 0.001	< 0.001
**DBP, mmHg**	81.1 (12.0)	82.2 (12.6)	79.5 (11.9)	< 0.001	< 0.001
**BMI, kg/m**^**2**^	21.5 (2.6)	22.0 (2.8)	21.0 (2.4)	< 0.001	< 0.001
**Laboratory results, mg/dl**					
Glucose	98.5 (91.8, 105.3)	97.2 (90.7, 104.4)	96.3 (89.8, 103.5)	< 0.001	< 0.001
Total cholesterol	171.7 (150.0, 192.6)	172.9 (154.3, 195.0)	168.0 (148.5, 189.5)	< 0.001	0.207
Triglycerides	86.8 (65.5, 119.8)	89.0 (70.0, 122.7)	82.4 (62.9, 113.4)	< 0.001	0.029
High density lipoprotein	53.6 (45.2, 64.3)	54.5 (45.6, 65.0)	54.5 (45.2, 65.4)	0.384	0.072
**Alcohol Status, No. (%)**				< 0.001	
Never	670 (67.7)	225 (42.6)	1947 (50.7)		
Former	17 (1.7)	40 (7.6)	98 (2.6)		
Current	303 (30.6)	263 (49.8)	1792 (46.7)		
**Education Level (%)**				< 0.001	
Illiterate	212 (21.5)	114 (21.7)	960 (25.0)		
Elementary school	457 (46.4)	270 (51.3)	1947 (50.8)		
Middle school and above	316 (32.1)	142 (27.0)	928 (24.2)		
**Occupation Type, farmer (%)**	874 (88.4)	477 (90.5)	3540 (92.1)	0.001	
**History of Hypertension, yes (%)**	62 (6.3)	48 (9.1)	146 (3.8)	< 0.001	
**History of Diabetes, yes (%)**	11 (1.1)	1 (0.2)	13 (0.3)	0.004	

*Abbreviations*: *BMI* body mass index, *DBP* diastolic blood pressure, *HDL* high-density lipoprotein, *SBP* systolic blood pressure. For continuous variables, values are presented as mean (SD) and mean (SE). Laboratory results are presented as median (IQR). *The differences between never smokers and the current smokers was corrected by the Bonferoni method

### Effects of smoking status on mortality

**Fig. 1:**
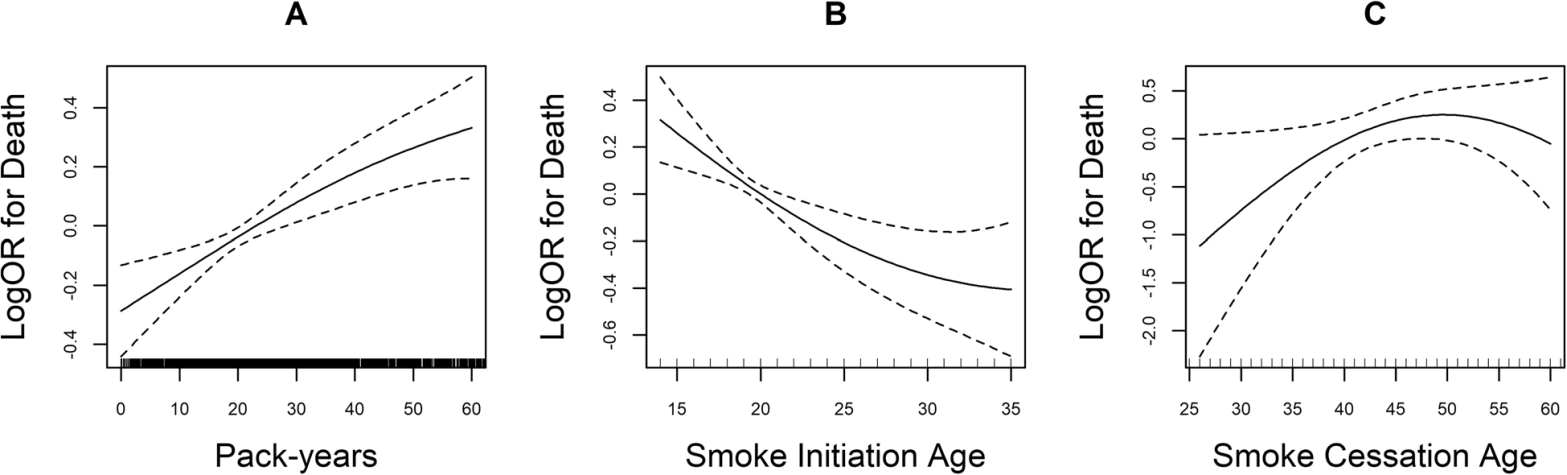
Smooth curves between smoking status and death

We observed a positive association between baseline pack-years and risk of death (logOR) after adjustment (Fig. 1a). Figure 1b and c show that younger smoking initiation age and older smoking cessation age were associated with a higher risk of death. The graph displays a decrease in mortality risk as smoking initiation age increases, and an increase in death risk as smoking cessation age increases. This finding was further evaluated by GEE models as shown in Table 2. Current cigarette smokers (odds ratio ([OR], 1.60; 95% CI, 1.23,2.08, *P* < 0.001) and former smokers ([OR], 2.12; 95%CI, 1.49,3.01, *P* < 0.001) had higher all-cause mortality risk than non-tobacco users. Relative to nonsmokers, current smokers of fewer than 20 pack-years ([OR], 1.18; 95%CI, 0.85,1.64, *P* = 0.314), 20 to 40 pack-years ([OR], 1.69; 95%CI, 1.28–2.23, *P* < 0.001) and more than 40 pack-years ([OR], 1.85; 95% CI, 1.33,2.56, *P* < 0.001) had a higher all-cause mortality risk.

**Table 2:** The association between smoking status and mortality in male

Variables	N	Deaths (%)	Crude model	***P*** value	Adjusted model^**a**^	***P*** value
			OR (95%CI)		OR (95%CI)	
**Smoking Status**						
Never	990	74 (7.5)	*ref*		*ref*	
Former	528	75 (14.2)	2.05 (1.46,2.88)	< 0.001	2.12 (1.49,3.01)	< 0.001
Current	3849	430 (11.2)	1.56 (1.20,2.01)	< 0.001	1.60 (1.23,2.08)	< 0.001
**Pack-years**						
Current						
< 20	960	82 (8.5)	1.16 (0.84,1.60)	0.378	1.18 (0.85,1.64)	0.314
20–40	2148	245 (11.4)	1.59 (1.22,2.09)	< 0.001	1.69 (1.28,2.23)	< 0.001
≥ 40	741	103 (13.9)	2.00 (1.46,2.74)	< 0.001	1.85 (1.33,2.56)	< 0.001

^a^Adjusted for age, body mass index, systolic blood pressure, diastolic blood pressure, fasting glucose, total cholesterol, triglycerides, high-density lipoprotein cholesterol and alcohol drinking status, education level and occupation

### Effects of smoking initiation age and smoking cessation age on mortality

For smoking initiation age, compared with nonsmokers, current smokers who began smoking before age 20 had a 91% ([OR], 1.91; 95%CI, 1.43,2.54; *P* < 0.001) increased risk of death, and those who began after age 20 ([OR],1.39; 95%CI, 1.06,1.84; *P* = 0.019), had a 39% increased risk of death. The results were consistent across the high and low pack-year groups (Table 3). Those whose smoking cessation age was older than 55 years had a 296% ([OR], 3.96; 95%CI, 1.03,15.14; *P* = 0.045) and 158% ([OR], 2.58; 95%CI, 1.18,5.63; *P* = 0.018) increased risk for death in the low vs. high pack-year groups, respectively (Table 4).

**Table 3:** The relation of smoking initiation age and pack-years with mortality in current smokers

Variables	N	Deaths (%)	Crude model	***P*** value	Adjusted model^**a**^	***P*** value
			OR (95%CI)		OR (95%CI)	
Never	990	74 (7.5)	*ref*		*ref*	
Age at smoking initiation, yrs						
B1(≥20)	2203	221 (10)	1.38 (1.05,1.81)	0.020	1.39 (1.06,1.84)	0.019
B2(< 20)	1646	209 (12.7)	1.80 (1.36,2.38)	< 0.001	1.91 (1.43,2.54)	< 0.001
Pack-years(< 20)						
≥ 20	697	59 (8.5)	1.14 (0.81,1.63)	0.452	1.22 (0.85,1.75)	0.279
< 20	263	23 (8.7)	1.19 (0.73,1.93)	0.491	1.26 (0.76,2.08)	0.378
Pack-years(20–40)						
≥ 20	1230	130 (10.6)	1.46 (1.09,1.97)	0.012	1.50 (1.11,2.04)	0.009
< 20	918	115 (12.5)	1.77 (1.30,2.41)	< 0.001	1.98 (1.43,2.74)	< 0.001
Pack-years(≥40)						
≥ 20	276	32 (11.6)	1.62 (1.05,2.51)	0.030	1.42 (0.90,2.23)	0.132
< 20	465	71 (15.3)	2.23 (1.58,3.14)	< 0.001	2.13 (1.49,3.06)	< 0.001

^a^Adjusted for age, body mass index, systolic blood pressure, diastolic blood pressure, fasting glucose, total cholesterol, triglycerides, high-density lipoprotein cholesterol and alcohol drinking status, education level and occupation

**Table 4:** The relation of smoking cessation age and pack-years with mortality in former smoker

Smoking Status	N	Deaths (%)	Crude		Adjusted^**a**^	
			OR (95%CI)	***P value***	OR (95%CI)	***P value***
Never	990	74 (7.5)	ref		ref	
Age at cessation, yrs						
< 55	465	59 (12.7)	1.80 (1.25,2.59)	0.002	2.20 (1.49,3.27)	< 0.001
≥ 55	63	16 (25.4)	4.21 (2.29,7.75)	< 0.001	2.22 (1.45,3.41)	0.004
pack-years (<median (26))						
< 55	246	32 (13.0)	1.85 (1.19, 2.88)	0.006	2.66 (1.63, 4.33)	< 0.001
≥ 55	10	4 (40.0)	8.25 (2.28,29.85)	0.001	3.96 (1.03,15.14)	0.045
pack-years (≥median (26))						
< 55	219	27 (12.3)	1.74 (1.09,2.78)	0.020	1.94 (1.18,3.20)	0.009
≥ 55	53	12 (22.6)	3.62 (1.84,7.13)	< 0.001	2.58 (1.18,5.63)	0.018

^a^Adjusted for age, body mass index, systolic blood pressure, diastolic blood pressure, fasting glucose, total cholesterol, triglycerides, high-density lipoprotein cholesterol and alcohol drinking status, education level and occupation

### The joint effect between drinking and smoking on mortality in males

After adjustment, we found that current drinkers who did not smoke had an insignificantly 42% ([OR], 0.58; 95%CI, 0.32,1.05; *P* = 0.072) decreased risk of death compared to nondrinkers who did not smoke. Those who were both former drinkers and former smokers ([OR], 6.40; 95%CI, 3.03, 13.50; *P* < 0.001) had an all-cause mortality risk of more than five times higher than nondrinkers who did not smoke. Regardless of drinking status, the ever-smoking population showed an upward trend in mortality (Table 5). We further performed stratified analyses to assess the effects of smoking status (never, former and current) on death in various subgroups (Supplemental Table 1).

**Table 5:** The joint effect between drinking and smoking on mortality in males

Drinking Status	Smoking Status					
	Never		Former		Current	
	Deaths (%)	OR (95%CI)	Deaths (%)	OR (95%CI)	Deaths (%)	OR (95%CI)
Never	58 (8.7)	*Ref*	35 (15.6)	2.18 (1.37, 3.48)	204 (10.5)	1.31 (0.95, 1.80)
Former	1 (5.9)	0.66 (0.09, 4.65)	14 (35.0)	6.40 (3.03,13.50)	22 (22.4)	2.56 (1.47, 4.47)
Current	15 (5)	0.58 (0.32, 1.05)	26 (9.9)	1.23 (0.74, 2.04)	203 (11.3)	1.39 (1.00, 1.93)

P for interaction = 0.034

Adjusted for age, body mass index, systolic blood pressure, diastolic blood pressure, fasting glucose, total cholesterol, triglycerides, high-density lipoprotein cholesterol, alcohol drinking status, education level and occupation

## Discussion

Our study provides further support that current and former smokers experienced greater risk of death at follow-up than nonsmokers in a Chinese rural population. Younger age at smoking initiation and older age at smoking cessation were both associated with increased risk of mortality.

Risk of all-cause mortality was significantly higher in former and current smokers than in nonsmokers. This finding is similar to the results from The National Longitudinal Mortality Study [22]. Even more powerful evidence was found in another study from the National Institutes of Health–AARP Diet and Health Study [23], with a mean (SD) follow-up of 6.6 (1.3) years, which reported a dose-dependent association between smoking fewer than 1 CPD (HR, 1.99; 95% CI, 1.76, 2.25) or, 1 to 10 CPD (HR, 2.60; 95% CI, 2.45,2.75) (cigarettes per day) at baseline with all-cause mortality. These findings are of particular importance for they report on the effects of low intensity smoking.

Our study results are striking in that relatively small differences in age at initiation were associated with strong differences in mortality risk 15 years later. A National Health Interview Survey showed that age at smoking initiation among current smokers was associated with risks of cardiovascular (OR:1.67) and smoking-related cancers (OR = 2.1) [10]. Relative to former smokers, the risk of mortality was lower in individuals who quit smoking at earlier ages. This finding is in agreement with the Sax Institute’s 45 and Up Study [16]. Smoking cessation remains beneficial even at age 50. The investigation, which was based on 489,066 participants aged ≥60 years from 22 population-based cohorts of the CHANCES Consportium, confirmed that for former smokers, excess mortality and risk advancement periods (RAPs) decreased with time since cessation, with RAPs of 3.9 (95% CI, 3.0, 4.7), 2.7 (95% CI,1.8, 3.6), and 0.7 (95% CI,0.2, 1.1) for those who had quit < 10, 10 to 19, and ≥ 20 years ago, respectively [24]. Similarly, the Zutphen Study found that in 1373 men, cessation of cigarette smoking at age 40 increased life expectancy by 4.6 years, while the number of disease-free life-years was increased by 3.0 years [25].

To date, most studies about the effects of smoking have focused on urban or general populations [26], while only limited studies on smoking and mortality risk have been carried out in Chinese rural areas and in particular, none have had such a long follow-up period or large prospective cohort among a male farming population as the current study. A consistent trait for all of these studies was that with the increase in pack-years in current smokers, all-cause mortality climbed remarkable, however our study showed contrary results in former smokers. It is likely that among men who had stopped smoking due to illness, the protective effects of quitting cannot be assessed straightforwardly, even if cessation is substantially protective, because the underlying illness that prompted the smoking cessation may cause a misleading, elevated risk. The question remains: how does tobacco use attribute to all-cause mortality? In a Chinese report on the health hazards of smoking, it was noted that tobacco smoke contains 69 known carcinogens, which can cause mutations in key genes, dysregulate normal growth control mechanisms, and eventually lead to the occurrence of cell cancer and malignant tumors [27]. In addition, it also damages vascular endothelial function, which can lead to the occurrence of atherosclerosis, narrowing of the arterial vascular cavity, and cause a variety of cardiovascular and cerebrovascular diseases.

Our study has two new findings. Firstly, the age of first tobacco use is an important determinant of mortality risk. Age at smoking initiation was strongly associated with mortality in men over 45 years of age. Ever smokers who started smoking earlier were at a progressively higher risk of mortality during follow-up, relative to those who started smoking later. Similarly, risk of mortality was lower when cessation occurred at an earlier age. This finding supports analogous results abroad. A possible reason is that an earlier age of smoking initiation and a later age of smoking cessation, increases duration of exposure. Secondly, our results suggest that nonsmokers who were ever drinkers, had a potent protective factor for mortality risk in this rural Chinese population, while, former smokers who were former drinkers were at risk. We surmise that the increase in mortality risk for those who previously used cigarettes and alcohol among this rural population could be a result of stopping usage due to illness. Because the cessation patterns in rural areas are different from those in urban areas, ordinary public health incentives may not effectively prevent smoking initiation and may fail to induce current smokers to quit. For example, indoor smoking bans may not have a substantial impact on middle-aged and elderly people in rural areas, because the majority of them are farmers who work out of doors. Raising the tobacco sales tax may only push rural residents to purchase cheaper cigarettes, because the cigarette prices in China vary greatly. Hence if interventions are adopted to suit the situations and demands for this population, the protection in rural villages will be more effective, particularly in the context of unbalanced social and economic development [11].

This study has specific strengths and limitations. Firstly, the data were sparse after stratification, resulting in a larger 95% confidence interval and insignificant results. Secondly, in rural China, smoking cessation was motivated mainly by health issues experienced either directly or indirectly. Nearly all participants who had attempted to quit or have successfully quit smoking, reported experiencing some health issues prior to quitting. Those who successfully quit frequently reported significant health events that prompted a visit to a doctor [28]. Lastly, only smoking information collected at baseline was available for this analysis, therefore, it is possible that recall bias existed and some participants who were former smokers at baseline may have resumed smoking afterward, leading to an underestimation of benefits related to smoking cessation. Another weakness of the study was the lack of classification and time of death. Data on smoking-attributable causes of death would have been informative in our interpretation of these results.

## Conclusions

We provide further evidence that cigarette smoking, regardless of amount, confers significant mortality risks, and that pack-years and age of smoking initiation and cessation, both key components of smoking duration, are important predictors of mortality in Chinese rural adults aged 45 years and older. Younger age at initiation was associated with increased risk of mortality, highlighting the seriousness of the impact that young adult smoking has on lifetime mortality risk. Therefore, smoking cessation incentives and the health benefits of nonsmoking should be promoted and emphasized to youth and all smokers, regardless of age.

## Supplementary Information

**Additional file 1: Figure S1.** Flowchart of the study participants.

**Additional file 2: Table S1.** Stratified analyses of risk factors on death by smoking status in males. **Table S2.** Baseline characteristics of the study participants by smoking status in females.

## Data Availability

The data supporting the findings of this study will be available from the author (Xiping Xu xipingxu126@126.com.) on request.

## Abbreviations

*SBP*: Systolic blood pressure
*DBP*: Diastolic blood pressure
*GEE*: Generalized estimating eqs.
*BMI*: Body mass index
*TC*: Total cholesterol
*TG*: Triglycerides
*HDL-C*: High-density lipoprotein cholesterol
*CI*: Confidence interval
*OR*: Odds ratio

## References

[1] Li S, Meng L, Chiolero A, Ma C, Xi B (2016). Trends in smoking prevalence and attributable mortality in China, 1991–2011. Prev Med.

[2] Chen Z, Peto R, Zhou M, Iona A, Smith M, Yang L, Guo Y, Chen Y, Bian Z, Lancaster G, Sherliker P, Pang S, Wang H, Su H, Wu M, Wu X, Chen J, Collins R, Li L, China Kadoorie Biobank (CKB) collaborative group (2015). Contrasting male and female trends in tobacco-attributed mortality in China: evidence from successive nationwide prospective cohort studies. Lancet.

[3] Yang G, Wang Y, Wu Y, Yang J, Wan X (2015). The road to effective tobacco control in China. Lancet.

[4] Liu S, Zhang M, Yang L, Li Y, Wang L, Huang Z, Wang L, Chen Z, Zhou M (2017). Prevalence and patterns of tobacco smoking among Chinese adult men and women: findings of the 2010 national smoking survey. J Epidemiol Community Health.

[5] China Global Adult Tobacco Survey 2018.

[6] Zhou M, Wang H, Zeng X, Yin P, Zhu J, Chen W, Li X, Wang L, Wang L, Liu Y, Liu J, Zhang M, Qi J, Yu S, Afshin A, Gakidou E, Glenn S, Krish VS, Miller-Petrie MK, Mountjoy-Venning WC, Mullany EC, Redford SB, Liu H, Naghavi M, Hay SI, Wang L, Murray CJL, Liang X (2019). Mortality, morbidity, and risk factors in China and its provinces, 1990–2017: a systematic analysis for the global burden of disease study 2017. Lancet.

[7] Kenfield SA, Stampfer MJ, Rosner BA, Colditz GA (2008). Smoking and smoking cessation in relation to mortality in women. JAMA.

[8] Nash SH, Liao LM, Harris TB, Freedman ND (2017). Cigarette smoking and mortality in adults aged 70 years and older: results from the NIH-AARP cohort. Am J Prev Med.

[9] Jha P, Ramasundarahettige C, Landsman V, Rostron B, Thun M, Anderson RN, McAfee T, Peto R (2013). 21st-century hazards of smoking and benefits of cessation in the United States. N Engl J Med.

[10] Choi SH, Stommel M (2017). Impact of age at smoking initiation on smoking-related morbidity and all-cause mortality. Am J Prev Med.

[11] Zhi K (2017). Trends in cigarette smoking among older male adults in China: an urban–rural comparison. J Appl Gerontol.

[12] Zhang J, Ou J-X, Bai C-X (2011). Tobacco smoking in China: prevalence, disease burden, challenges and future strategies. Respirology.

[13] Rich ZC, Hu M, Xiao S (2014). Gifting and sharing cigarettes in a rural Chinese village: a cross-sectional study. Tob Control.

[14] Lariscy JT, Hummer RA, Rogers RG (2018). Cigarette smoking and all-cause and cause-specific adult mortality in the United States. Demography.

[15] Cho MH (2018). Effects of smoking habit change on all-cause mortality and cardiovascular diseases among patients with newly diagnosed diabetes in Korea. Sci Rep.

[16] Banks E, Joshy G, Weber MF, Liu B, Grenfell R, Egger S, Paige E, Lopez AD, Sitas F, Beral V (2015). Tobacco smoking and all-cause mortality in a large Australian cohort study: findings from a mature epidemic with current low smoking prevalence. BMC Med.

[17] Feng Y, Hsu YH, Terwedow H, Chen C, Xu X, Niu T, Zang T, Wu D, Tang G, Li Z, Hong X, Wang B, Brain JD, Cummings SR, Rosen C, Bouxsein ML, Xu X (2005). Familial aggregation of bone mineral density and bone mineral content in a Chinese population. Osteoporos Int.

[18] Hsu YH, Venners SA, Terwedow HA, Feng Y (2006). Relation of body composition, fat mass, and serum lipids to osteoporotic fractures and bone mineral density in Chinese men and women1–3. Am J Clin Nutr.

[19] Yi-Hsiang Hsu XX, Terwedow HA (2006). A large-scale genome-wide linkage analysis for loci linked to bone mineral density. J Bone Miner Res.

[20] 中国临床戒烟指南 (2015年版)

[21] Agudo A, Ahrens W, Benhamou E, Benhamou S, Boffetta P, Darby SC, Simonato L (2000). Lung cancer and cigarette smoking in women: a multicenter case-control study in Europe. Int J Cancer.

[22] Christensen CH, Rostron B, Cosgrove C, Altekruse SF, Hartman AM, Gibson JT, Apelberg B, Inoue-Choi M, Freedman ND (2018). Association of cigarette, cigar, and pipe use with mortality risk in the US population. JAMA Intern Med.

[23] Inoue-Choi M, et al. Association of long-term, low-intensity smoking with all-cause and cause-specific mortality in the National Institutes of Health–AARP Diet and Health Study. JAMA Intern Med. 2017;177:87–95. 10.1001/jamainternmed.2016.7511 PMC5555224 27918784

[24] Mons U, Muezzinler A, Gellert C, Schottker B, Abnet CC, Bobak M, de Groot L, Freedman ND, Jansen E, Kee F, Kromhout D, Kuulasmaa K, Laatikainen T, O'Doherty MG, Bueno-de-Mesquita B, Orfanos P, Peters A, van der Schouw YT, Wilsgaard T, Wolk A, Trichopoulou A, Boffetta P, Brenner H, on behalf of the CHANCES consortium (2015). Impact of smoking and smoking cessation on cardiovascular events and mortality among older adults: meta-analysis of individual participant data from prospective cohort studies of the CHANCES consortium. BMJ.

[25] Streppel MT, Boshuizen HC, Ocke MC, Kok FJ, Kromhout D (2007). Mortality and life expectancy in relation to long-term cigarette, cigar and pipe smoking: the Zutphen study. Tob Control.

[26] Lam TH, Xu L, Jiang CQ, Zhang WS, Zhu F, Jin YL, Thomas GN, Cheng KK (2018). High relative risk of all-cause mortality attributed to smoking in China: Guangzhou biobank cohort study. PLoS One.

[27] 中华人民共和国卫生部 (2012). 中国吸烟危害健康报告.

[28] Jiang Y, Elton-Marshall T, Fong GT, Li Q (2010). Quitting smoking in China: findings from the ITC China survey. Tob Control.

