# Supplementary figures and images for: Cigarette smoking and all-cause mortality in rural Chinese male adults: 15-year follow-up of the Anqing cohort study
Source: BMC Public Health. 2021 Apr 9;21:696. doi: 10.1186/s12889-021-10691-2 (PMC8034075; doi:10.1186/s12889-021-10691-2)

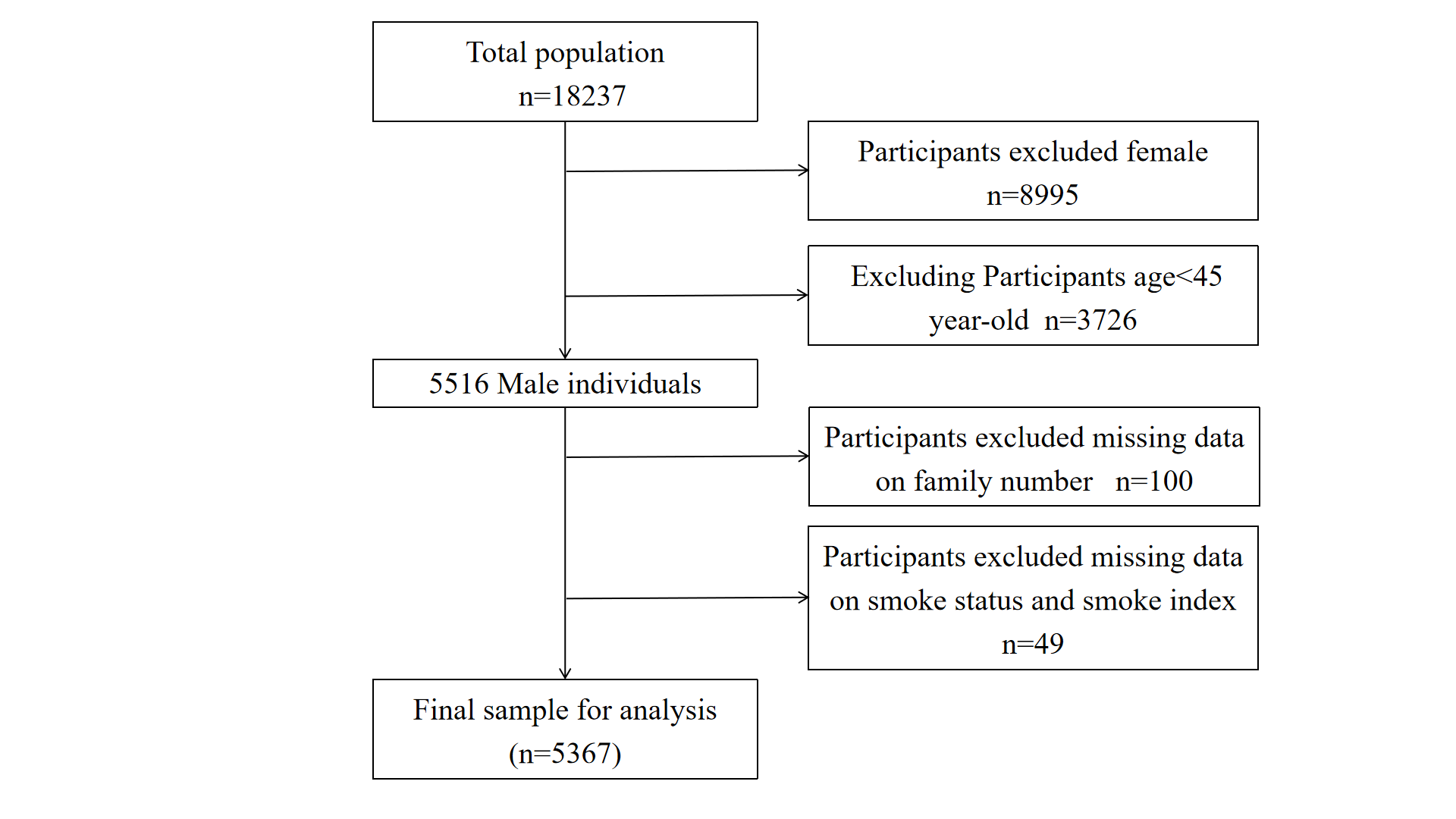

Supplement: Supplementary file 1 — Additional file 1: Figure S1. Flowchart of the study participants. [file 12889_2021_10691_MOESM1_ESM.tif]
