# Supplementary material for: Cigarette smoking and all-cause mortality in rural Chinese male adults: 15-year follow-up of the Anqing cohort study
Source: BMC Public Health. 2021 Apr 9;21:696. doi: 10.1186/s12889-021-10691-2 (PMC8034075; doi:10.1186/s12889-021-10691-2)
Supplement: Supplementary file 2 — Additional file 2: Table S1. Stratified analyses of risk factors on death by smoking status in males. Table S2. Baseline characteristics of the study participants by smoking status in females. [file 12889_2021_10691_MOESM2_ESM.docx]

**Supplemental Table 1. Stratified analyses of risk factors on death in males by smoking status in male**

| **Variables** | **Never** | | **Former** | | | **Current** | | | ***P*** for interaction |
| --- | --- | --- | --- | --- | --- | --- | --- | --- | --- |
|  | **N** | **Deaths (%)** | **N** | **Deaths (%)** | **OR (95%CI)** | **N** | **Deaths (%)** | **OR (95%CI)** |  |
| **Age, years** |  |  |  |  |  |  |  |  | 0.489 |
| <51.3 | 484 | 29 (6) | 242 | 24 (9.9) | 1.87 (1.05,3.33) | 1957 | 151 (7.7) | 1.30 (0.85,1.97) |  |
| ≥51.3 | 506 | 45 (8.9) | 286 | 51 (17.8) | 2.31 (1.47,3.61) | 1892 | 279 (14.7) | 1.80 (1.28,2.54) |  |
| **Systolic Blood Pressure, mmHg** | | |  |  |  |  |  |  | 0.891 |
| <130 | 588 | 38 (6.5) | 293 | 38 (13) | 2.32 (1.43,3.76) | 2543 | 242 (9.5) | 1.57 (1.10,2.23) |  |
| 130-140 | 165 | 15 (9.1) | 79 | 10 (12.7) | 1.70 (0.70,4.13) | 524 | 69 (13.2) | 1.43 (0.77,2.64) |  |
| ≥140 or history of hypertension | 237 | 21 (8.9) | 156 | 27 (17.3) | 2.20 (1.17,4.16) | 777 | 119 (15.3) | 1.77 (1.06,2.94) |  |
| **Diastolic Blood Pressure, mmHg** | | |  |  |  |  |  |  | 0.739 |
| <80 | 431 | 34 (7.9) | 225 | 26 (11.6) | 1.71 (0.98,2.99) | 1958 | 199 (10.2) | 1.38 (0.94,2.02) |  |
| 80-90 | 309 | 22 (7.1) | 155 | 23 (14.8) | 2.65 (1.41,4.97) | 1117 | 122 (10.9) | 1.65 (1.03,2.64) |  |
| ≥90 or history of hypertension | 250 | 18 (7.2) | 148 | 26 (17.6) | 2.44 (1.23,4.84) | 768 | 108 (14.1) | 1.89 (1.08,3.31) |  |
| **BMI ,kg/m2** |  |  |  |  |  |  |  |  | 0.594 |
| T1(<20.0) | 278 | 25 (9) | 127 | 27 (21.3) | 2.96 (1.6,5.46) | 1368 | 197 (14.4) | 1.86 (1.20,2.88) |  |
| T2(20.0-21.9) | 323 | 26 (8) | 161 | 21 (13) | 1.56 (0.85,2.89) | 1305 | 126 (9.7) | 1.21 (0.78,1.89) |  |
| T3(≥21.9) | 387 | 22 (5.7) | 238 | 27 (11.3) | 2.01 (1.11,3.66) | 1157 | 107 (9.2) | 1.82 (1.13,2.94) |  |
| **Alcohol** Drinking Status |  |  |  |  |  |  |  |  | 0.034 |
| Never | 670 | 58 (8.7) | 225 | 35 (15.6) | 2.18 (1.37,3.47) | 1947 | 204 (10.5) | 1.26 (0.91,1.74) |  |
| Former | 17 | 1 (5.9) | 40 | 14 (35) | 17.90 (3.12,102.78) | 98 | 22 (22.4) | 5.85 (1.23, 27.82) |  |
| Current | 303 | 15 (5) | 263 | 26 (9.9) | 2.09 (1.07,4.06) | 1792 | 203 (11.3) | 2.45 (1.43,4.19) |  |

Adjusted for age, body mass index, systolic blood pressure, diastolic blood pressure, fasting glucose, total cholesterol, triglycerides, high-density lipoprotein cholesterol and alcohol drinking status, education level and occupation . CI indicates confidence interval;

**Supplemental Table 2. Baseline characteristics of the study participants by smoking status in female**

| **Variables** | **Smoking Status** | | | ***P* value** |
| --- | --- | --- | --- | --- |
|  | **Never** (n=4027) | **Former** (n=19) | **Current** (n=216) |  |
| **Age, y** | 51.2 (4.4) | 52.8 (3.4) | 51.7 (4.3) | 0.081 |
| **SBP, mmHg** | 126.8 (21.2) | 127.6 (21.1) | 119.2 (20.6) | <0.001 |
| **DBP, mmHg** | 78.9 (11.8) | 81.7 (13.4) | 75.6 (12.2) | <0.001 |
| **BMI, kg/m^2^** | 22.1 (3.5) | 23.1 (3.3) | 21.4 (2.8) | 0.004 |
| **Laboratory results, mg/dl** |  |  |  |  |
| Glucose | 98.1 (91.8, 105.8) | 94.7 (91.8, 100.8) | 95.0 (90.3, 103.7) | 0.003 |
| Total cholesterol | 176.7 (156.2, 199.5) | 160.5 (147.2, 196.7) | 172.1 (155.5, 192.3) | 0.094 |
| Triglycerides | 109.8 (84.1, 148.8) | 102.3 (75.5, 136.8) | 113.4 (82.8, 148.8) | 0.840 |
| High density lipoprotein | 53.0 (44.9, 62.6) | 51.6 (48.8, 57.2) | 52.2 (45.6, 61.5) | 0.871 |
| **Alcohol Status, No. (%)** |  |  |  | <0.001 |
| Never | 3923 (97.5) | 14 (73.7) | 174 (80.9) |  |
| Former | 15 (0.4) | 2 (10.5) | 4 (1.9) |  |
| Current | 84 (2.1) | 3 (15.8) | 37 (17.2) |  |
| **Education Level, No. (%)** |  |  |  | 0.113 |
| Illiterate | 3229 (80.7) | 14 (77.8) | 173 (80.1) |  |
| Elementary school | 644 (16.1) | 4 (22.2) | 42 (19.4) |  |
| Middle school and above | 129 (3.2) | 0 (0.0) | 1 (0.5) |  |
| **Occupation Type=farmer (%)** | 3780 (94.3) | 17 (89.5) | 201 (93.1) | 0.500 |
| **History of Hypertension, yes(%)** | 221 ( 5.5) | 2 (10.5) | 12 (5.6) | 0.631 |
| **History of Diabetes, yes(%)** | 26 (0.6) | 0 (0.0) | 1 (0.5) | 0.891 |

Abbreviations: BMI, body mass index; DBP, diastolic blood pressure; HDL, high-density lipoprotein; SBP, systolic blood pressure. *For continuous variables, values are presented as mean (SD) and mean (SE).. Laboratory results are presented as median (IQR).
